# Real Life Efficacy and Safety of Secukinumab in Biologic-Experienced Patients With Psoriatic Arthritis

**DOI:** 10.3389/fmed.2020.00288

**Published:** 2020-06-19

**Authors:** Kalliopi Klavdianou, Argyro Lazarini, Alexandros Grivas, Dimitrios Tseronis, Christina Tsalapaki, Panagiota Rapsomaniki, Katerina Antonatou, Konstantinos Thomas, Dimitrios Boumpas, Pelagia Katsimbri, Dimitrios Vassilopoulos

**Affiliations:** ^1^Clinical Immunology-Rheumatology Unit, 2nd Department of Medicine and Laboratory, Hippokration General Hospital, Athens, Greece; ^2^Clinical Immunology-Rheumatology Unit, 4th Department of Medicine, School of Medicine, Attikon University Hospital, National and Kapodistrian University of Athens, Athens, Greece

**Keywords:** psoriatic arthritis, secukinumab, tumor necrosis factor inhibitors, biologic agents, real world evidence, ustekinumab

## Abstract

**Background:** Real world evidence data regarding secukinumab (SEC) use in biologic-experienced patients with psoriatic arthritis (PsA) are scarce.

**Objectives:** To assess the real life survival, safety and efficacy of SEC in biologic-experienced patients with PsA.

**Methods:** All biologic-experienced PsA patients treated with SEC in 2 University Rheumatology Units were included (3/2016-12/2018). Patients' and disease characteristics were recorded at baseline and during SEC therapy.

**Results:** 75 patients were included; 76% were females with a mean age of 53.9 years, median disease duration of 6.7 years and median SEC treatment duration of 11.1 months. At baseline, 97% had peripheral arthritis, 42% axial involvement, 22% enthesitis, and 12% dactylitis. Regarding previous biologic exposure, 48 (64%) had been exposed to anti-tumor necrosis factor (TNF) agents only, 5 (7%) to the interleukin (IL)-12/23 inhibitor (Ustekinumab-UST) only while 22 (29%) both to anti-TNFs and UST. Fifty-three percent received SEC in combination with non-biologics and 35% with glucocorticoids, respectively. During follow-up, statistically significant improvement in different disease activity indices were noted (DAS28-CRP, DAPSA, BASDAI). SEC survival rate at the end of follow-up was 64% (48/75), without difference between patients exposed to anti-TNFs only (67%) vs. anti-TNFs and UST (68%) as well as to 1 vs. ≥2 anti-TNFs. The rate of serious adverse events and serious infections during follow-up was 4.8 and 1.2/100 patient-years, respectively.

**Discussion:** In real life, in biologic-experienced patients with PsA, SEC displayed a high retention rate, regardless of the type, and number of previous biologics (anti-TNFs ± anti-IL12/23), without significant side effects.

## Introduction

Secukinumab (SEC) is a recombinant human monoclonal antibody against interleukin-17A (IL17A) that has been approved since 2015 by the European Medicines Agency (EMA) and 2016 by the US Food and Drug Administration (FDA), for the treatment of psoriatic arthritis (PsA) (1, 2). Its efficacy and safety have been demonstrated in the randomized controlled trials (RCTs) that led to its approval (3–6) as well as in their extension studies (7–9).

Until recently, three different classes of biologics had been approved for PsA: the anti-tumor necrosis factor (TNF), the anti-IL12/23 (Ustekinumab—UST) and the anti-IL17 (SEC, ixekizumab) agents (10, 11). In RCTs, SEC has shown efficacy both in anti-TNF naïve as well as anti-TNF-experienced patients (3, 5, 6). So far, real world evidence (RWE) data regarding survival as well as efficacy and safety of SEC in patients with PsA are scarce (12–14), especially for those who had been exposed to both classes of biologics (anti-TNF, anti-IL12/23).

Thus, the aim of our study was to evaluate the use of SEC in biologic-experienced patients with PsA in daily clinical practice.

## Patients and Methods

This was a retrospective, observational, study of patients with PsA attending the Outpatient Rheumatology Clinics of two referral University Hospital Rheumatology Units. All included patients fulfilled the Caspar Classification Criteria (15) for PsA and had initiated treatment with SEC during the study period (March 2016—December 2018), according to their caring physicians' decision.

At baseline and during follow-up (every 3–6 months), data regarding patient (sex, age, gender, body mass index-BMI, smoking status, presence of co-morbidities according to the Rheumatic Diseases Comorbidity Index-RDCI) (16) and disease (disease duration, presence of enthesitis, dactylitis, or axial involvement, disease activity as measured by the Disease Activity in Psoriatic Arthritis Score—DAPSA (17), the Disease Activity Score—DAS28-CRP and Bath Ankylosing Spondylitis Disease Activity Index—BASDAI scores and function as measured by the health Assessment Questionnaire—HAQ and Bath Ankylosing Spondylitis Functional Index—BASFI scores, the percentage of skin disease assessed by the involved Body Surface Area—BSA) characteristics as well as treatment patterns (previous and current treatments including glucocorticoids, non-biologic, and biologics) were recorded for each patient. Furthermore, the number of patients who developed adverse events (AEs) or discontinued treatment were estimated. Reasons for discontinuation were classified as due to primary or secondary inefficacy, adverse events, or other.

All patients received the approved SEC dose of 300 mg subcutaneously (initially every week for the first 4 weeks and then every month). SEC was administered at the scheduled time of their previous bDMARD administration.

Study approval was provided by the local institutional boards of the two participating centers.

### Statistical Analysis

Descriptive analysis was initially performed. Categorical data were presented as counts and percentage of patients in each category. Continuous data were presented as mean ± standard deviation (S.D.) if normally distributed and median and interquartile range otherwise. The significance of difference in values obtained at baseline and last follow-up visit was assessed by Wilcoxon test for the continuous variables which were not normally distributed and chi-squared or Fisher's test for categorical variables.

The long-term survival of SEC was calculated until drug discontinuation or patient censoring and presented using Kaplan-Meier survival curves. Differences in drug survival between subgroups of patients were assessed using Log-rank test. Cox regression was used to evaluate predictive factors for drug discontinuation and hazard ratios (HRs) for discontinuation were calculated with univariate and multivariate models. The rates of adverse events and infections were calculated as events per 100 patient-years of follow-up. All statistical tests were two-sided and a *p* < 0.05 was considered statistically significant. Statistical analysis was performed using IBM SPSS Statistics, version 20.

## Results

### Baseline Characteristics

The baseline characteristics of the included patients are summarized in Table 1. All patients had been previously exposed to biologics; 64% (48/75) to anti-TNFs only, 7% (5/75) to UST only while 29% (22/75) had experienced both classes of biologics. Among anti-TNF ever exposed patients (*n* = 70), 49 (70%) had received ≥ 2 anti-TNF agents. During SEC therapy (Table 1), approximately half of the patients were co-treated with non-biologics (40/75, 53%) and ~ one third with glucocorticoids (35%).

**Table 1:** Patients and disease characteristics.

**Characteristics**	***n* with data available**	
*n*		75
Age, years, mean ± 1 *SD* (median)	75	53.9 ± 12.6 (50.7)
Females, *n* (%)	75	57 (76%)
Disease duration, years, mean ± 1 S.D. (median)	75	8.1 ± 6.9 (6.7)
Secukinumab treatment duration, months, mean ± 1 S.D. (median)	75	12.8 ± 8.9 (11.1)
Follow-up, months, mean ± 1 S.D. (median)	75	13 ± 9 (11.7)
**CLINICAL FINDINGS**		
Peripheral arthritis, *n* (%)	72	70 (97%)
Axial involvement, *n* (%)	74	31 (42%)
Enthesitis, *n* (%)	73	16 (22%)
Dactylitis, *n* (%)	74	9 (12%)
**COMORBIDITIES**		
Cardiovascular disease, *n* (%)	59	8 (14%)
Hypertension, *n* (%)	59	21 (36%)
Pulmonary disease, *n* (%)	58	11 (19%)
Diabetes, *n* (%)	59	11 (19%)
Depression, *n* (%)	59	7 (12%)
Smokers, *n* (%)	53	24 (45%)
BMI, mean ± 1 S.D. (median)	45	30.4 ± 7.6 (28.4)
RDCI, mean ± 1 S.D. (median)	72	1.57 ± 1.72 (1.0)
**PREVIOUS TREATMENTS**		
Anti-TNFs (ever)	75	70 (93%)
1	75	21 (28%)
2	75	29 (39%)
≥3	75	20 (27%)
Anti-TNFs (only)	75	48 (64%)
Ustekinumab (ever)	75	27 (36%)
Ustekinumab (only)	75	5 (7%)
Anti-TNFs and Ustekinumab	75	22 (29%)
Apremilast	75	5 (7%)
**CONCURRENT TREATMENT**		
Non-biologics, *n* (%)	75	40 (53%)
Methotrexate, *n* (%)	75	33 (44%)
Methotrexate dose (mg/week), mean ± 1 *SD*	33	15 ± 3
Leflunomide, *n* (%)	75	2 (3%)
Cyclosporine	75	1 (1%)
Glucocorticoids	75	26 (35%)
Prednisolone dose (mg/day), mean ± 1 *SD*	26	6.9 ± 7.7

*SD, Standard Deviation; BMI, Body Mass Index; RDCI, Rheumatic Diseases Co-morbidity Index; TNF, tumor necrosis factor*.

### Efficacy

**Figure 1:**
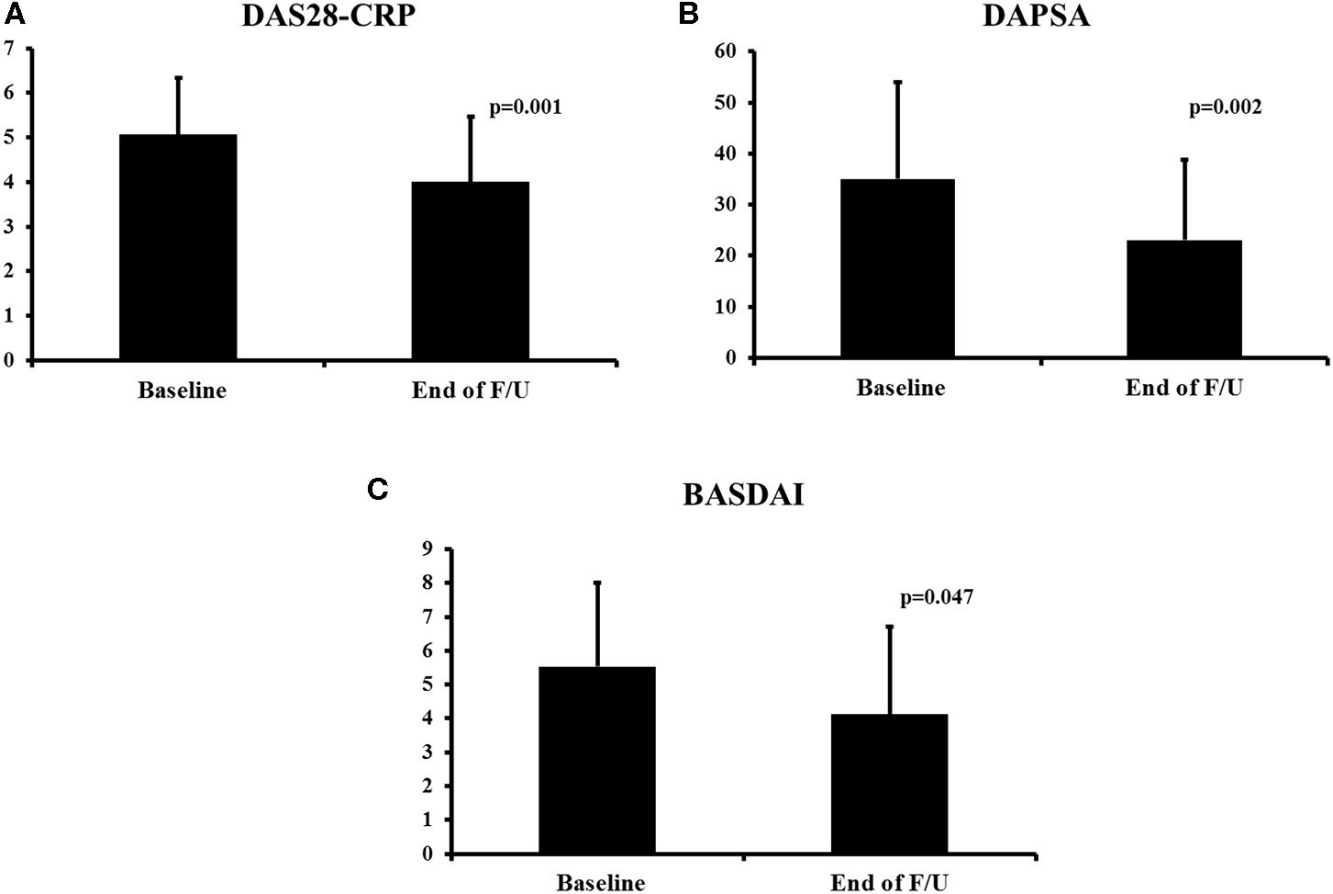
Change in disease activity indices during secukinumab therapy. DAS28-CRP **(A)**, DAPSA **(B)**, and BASDAI **(C)**. The mean ± *SD* of the respective values at baseline and last visit are shown. DAS28-CRP, Disease Activity Score28- C-Reactive Protein; BASDAI, Bath Ankylosing Spondylitis Disease Activity Index; F/U, follow-up.

At the end of the follow–up, improvement was noted in several disease indices. Peripheral arthritis as assessed by the DAS28-CRP and the DAPSA scores improved significantly (from a mean of 5.05 to 3.99, *p* = 0.001, Figure 1A and from 35.06 to 23.02, *p* = 0.002, Figure 1B, respectively). Similarly, there was improvement in patients' function as assessed by the HAQ score (from a mean of 0.79 to 0.56, *p* = 0.071). Dactylitis resolved in all patients who had it at baseline (*n* = 9, *p* = 0.003, Table 1) while enthesitis resolved in 56% of patients during follow-up (from 16/73, 22% at baseline to 7/73, 10% at last visit, *p* = 0.043).

For patients with axial involvement, there was a statistically significant improvement in the BASDAI score (from a mean of 5.53 to 4.12, *p* = 0.047, Figure 1C) and an improvement in the BASFI score (from a mean of 6.17 to 4.39, *p* = 0.141). In terms of the skin disease, the BSA score also decreased significantly (from a mean of 3.0 to 1.3, *p* = 0.001).

### Safety

Secukinumab was generally well-tolerated. Overall, nine patients (12%) experienced 11 AEs (13.3/100 patient-years); among those, 4 (5%) were considered serious (SAEs) leading to drug discontinuation at a rate of 4.8/100 patient-years during the follow-up period. These included two neoplasms at a rate of 2.4/100 patient-years (one colon cancer diagnosed in an 81 years old male 3 months and one case of endometrial cancer in a 45 years old female diagnosed 6 months, after SEC initiation, respectively), one case of serious soft tissue infection (requiring IV antibiotics) and one case of proteinuria (due to C3 glomerulopathy). There was one death in the 81 years old male patient who was diagnosed post-mortem with colon cancer (due to peritonitis).

Infections were uncommon (6/75, 8%) and in most cases (5/6) not serious. There were three opportunistic infections; two fungal (one oral-genital, one genital) treated only with local anti-fungals and one case of herpes zoster. The other three, included one case of serious periodontitis and two non-serious soft tissue infections. Only one infection was considered serious (soft tissue infection, requiring intravenous antibiotics) that led to drug discontinuation (overall rate of SIEs: 1.2/100 patient-years).

None of our patients had IBD at baseline and we did not observe any *de novo* cases of IBD during SEC treatment in our patient population.

### Drug Survival

**Figure 2:**
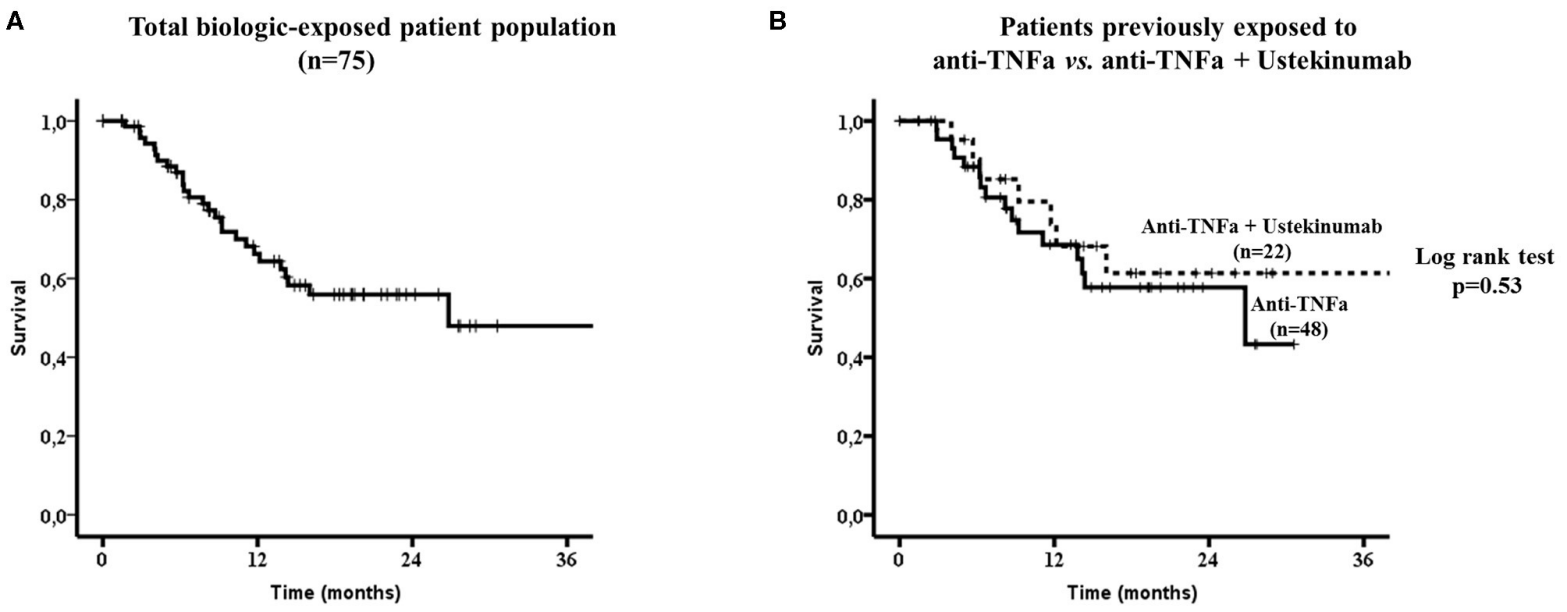
Secukinumab survival in the whole biologic-exposed patient population (*n* = 75, **A**) and to those exposed only to anti-TNFa (*n* = 48) vs. those exposed both to anti-TNFa and Ustekinumab (*n* = 22) **(B)**. No statistically significant difference in drug survival was noted between the two subgroups by the Log-rank test (*p* = 0.53). TNFa, tumor necrosis factor; IL12/23, interleukin12/23.

The overall drug survival at the last follow-up visit was 64% (48/75) with an estimated median drug survival of 26.8 months (Figure 2A). The estimated 1 and 2 years drug survival was 66% and 56%, respectively.

**Figure 3:**
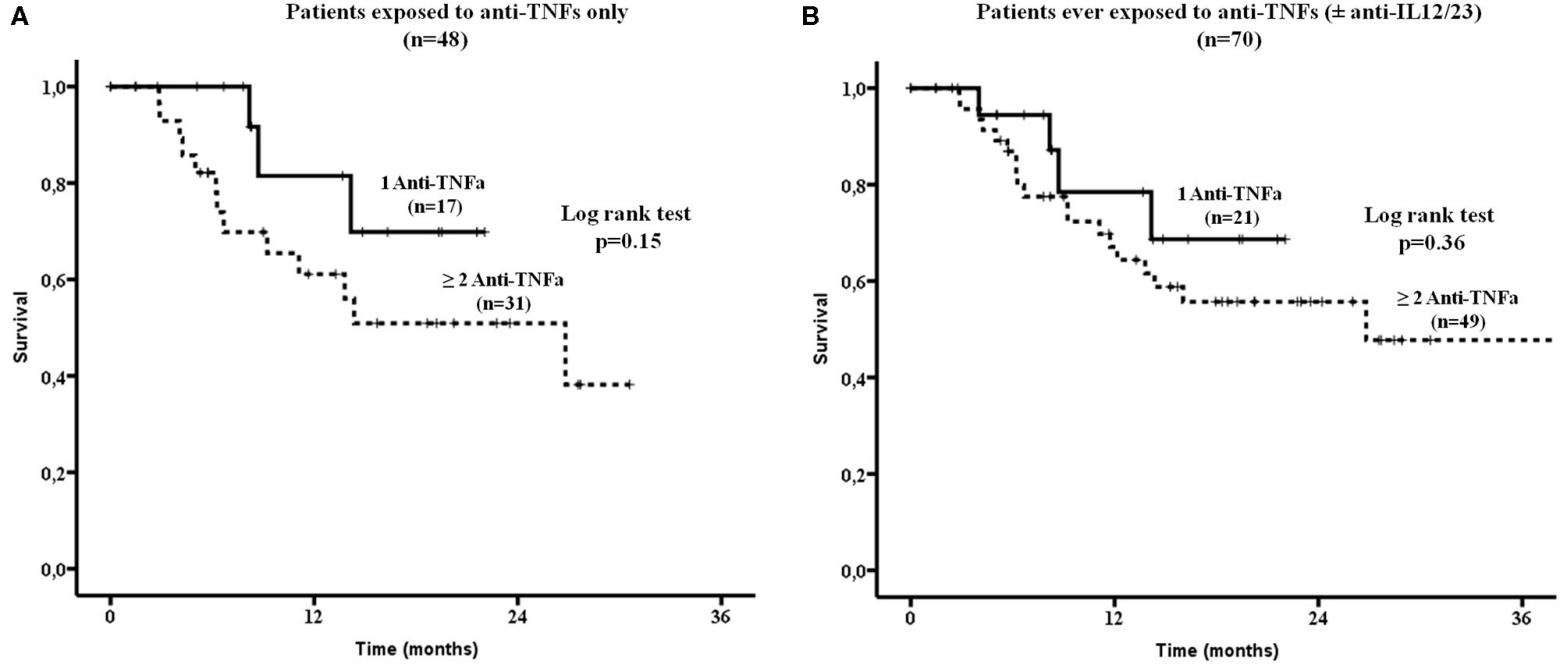
Secukinumab survival was estimated in patients exposed: **(A)** Only to anti-TNFa (*n* = 48). There was no statistically significant difference in drug survival between patients exposed to 1 vs. ≥ 2 anti-TNFa (by log rank test, *p* = 0.15) and **(B)** To anti-TNFa and/or Ustekinumab (anti-IL12/23, *n* = 70). Again no statistically significant difference in drug survival was noted between those ever exposed to 1 vs. ≥ 2 anti-TNFa (by log rank test, *p* = 0.36). TNFa, tumor necrosis factor; IL12/23, interleukin12/23.

There were no difference in drug survival between patients who had been exposed to anti-TNFs only (32/48, 67%) compared to those who had failed both anti-TNFs and anti-IL12/23 (15/22, 68%, Figure 2B). Although, patients who had been previously exposed to one anti-TNF had overall a better SEC survival compared to those exposed to ≥2 anti-TNFs, this did not reach statistical significance (Figures 3A,B, respectively).

The most common reasons for drug discontinuation (*n* = 27, 36%) was inadequate response (70%, 19/27, 10 due to primary and nine due to secondary lack of response), followed by adverse events (15%, 4/27) or other reasons (15%, 4/27).

No specific baseline or on-treatment factors were associated with SEC discontinuation by uni- and multivariate Cox regression analysis (Table S1).

## Discussion

This is one of the few real world studies assessing the usefulness of SEC in patients with PsA who had previously exposed to two different classes of biologics (anti-TNFs, anti-IL12/23). In this treatment-resistant group, the drug survival of SEC at the end of the follow-up was 64% with an acceptable safety profile similar to what has been previously reported in RCTs.

RCTs and their long term extension studies remain the gold-standard assessing the efficacy and safety of new therapies. On the other hand, real life cohorts provide valuable data regarding the overall safety, efficacy and drug survival of an approved medication in heterogeneous patient populations with various co-morbidities who usually are not registered in RCTs. Our real life patient population had some unique characteristics compared to the RCTs. This was a biologic-experienced, treatment-resistant population since almost all patients had been previously exposed to anti-TNFs (93%) and ~29% both to anti-TNF and anti-IL12/23 agents. Furthermore, these patients had long-standing, established disease (median: 6.7 years) and were recruited from University referral hospitals.

In the 3 RCTs, where a similar to our study SEC dose was used (300 mg), approximately one third of patients (31%) were anti-TNF-experienced (3, 5, 6). In this patient subgroup, the overall response rate at 24 weeks as assessed by the ACR-50 response criteria ranged from 23 to 41% (3, 5, 6). In our cohort, the peripheral joint disease activity (measured by the DAPSA and DAS28-CRP composite indices) as well as the skin disease (assessed by the BSA), improved significantly during SEC therapy. We also noted a statistically significant improvement in dactylitis and enthesitis in the studied population while a similar trend was seen in patients with axial involvement.

Regarding SEC survival, the estimated 1 and 2 years drug survival was 66% and 56%, respectively. So far, long term data regarding SEC survival in daily clinical practice are limited (12, 14, 18, 19). In a recent US study, Oelke et al. analyzed a claims database of 255 PsA patients treated with SEC (12). Among them, 163 patients had been previously exposed to biologics (anti-TNFs: 72%, anti-IL12/23: 45%). The overall 1 year survival rate of SEC in biologic-experienced patients was similar to our study (61% vs. 66% respectively), although no detailed data were given regarding the specific characteristics of these patients (12).

Compared to SEC, multiple patient registries and observational cohort studies have examined the survival of different anti-TNFs in biologic-experienced (mainly anti-TNFs) patients. In these studies, the 1 year drug survival ranged widely from 51 to 77% (12, 20–27). Nevertheless, real life data comparing directly anti-TNF and anti-IL17 (SEC) drug survival are missing. As mentioned above, Oelke et al. showed that the 1 year survival rate in biologic-experienced PsA patients treated with SEC was 61% compared with 53% in anti-TNF treated patients (12).

A novel finding of our study, was that for the first time we presented detailed data for patients with PsA treated with SEC who had been exposed both to anti-TNF and anti-IL12/23 agents. In our study, the overall SEC survival of patients exposed to both classes was not different from those exposed to anti-TNFs alone (68% vs. 67%). In contrast, to studies with anti-TNFs (22, 23, 25), we did not identify any baseline or on-treatment factors that correlated with SEC survival.

The safety profile of SEC was not much different to what has been previously reported in RCTs and their long-term extension studies (3, 5, 6, 9). The overall rate of SAEs was 4.8/100 patient-years which is lower to the rate (7.9/100 patient-years) reported in the long-term extension studies (9). Regarding serious infections, the overall incidence rate was 1.2/100 patient-years which was slightly lower to the 1.9/100 patient-years rate reported in the long term extension studies (9) and in a recent US claims database study (1.9/100 patient-years) (28). In this database study, the comparative incidence of serious infections in biologic-experienced patients treated with anti-TNFs or anti-IL12/23 were 2.7 and 1.7/100 patient-years, respectively (28).

We observed two fungal infections at a rate of 2.4/100 patient-years which is slightly higher to that of the long term extension studies (1.5/100 patient-years) (9). Both patients were treated with topical anti-fungals without interrupting SEC therapy. In our cohort, two malignancies were diagnosed at a rate of 2.4/100 patient-years which was higher to that reported by Deodhar et al. (1.1/100 patient-years) (9). It should be noted though that both neoplasms were diagnosed shortly after SEC initiation (first 6 months) and were considered unrelated to drug administration.

There are certain limitations to our study including its retrospective design, the potential missing data regarding AEs, the short period of follow-up and the absence of a control group of patients treated at the same period with different classes of biologics (anti-TNFs, anti-IL12/23).

In conclusion, we report one of the few real life studies in the literature assessing the survival as well as the safety and efficacy of SEC in patients with PsA who had been previously exposed to one or two different classes of biologics (anti-TNFs, anti-IL12/23). Our findings suggest that SEC is an efficacious and well-tolerated biologic agent in this multi-resistant, difficult to treat patient group.

## Data Availability Statement

The raw data supporting the conclusions of this article will be made available by the authors, without undue reservation.

## Ethics Statement

The studies involving human participants were reviewed and approved by Hippokration General Hospital, Athens, Greece Attikon University Hospital, Athens, Greece. The patients/participants provided their written informed consent to participate in this study.

## Author Contributions

KK, AL, PK, and DV: design of the study, data analysis, and manuscript preparation. KK, AL, AG, DT, CT, PR, KA, KT, DB, PK, and DV: acquisition of data. All authors reviewed the manuscript and gave final approval for the work.

## Conflict of Interest

The authors declare that the research was conducted in the absence of any commercial or financial relationships that could be construed as a potential conflict of interest.
