# Supplementary material for: Real Life Efficacy and Safety of Secukinumab in Biologic-Experienced Patients With Psoriatic Arthritis
Source: Front Med (Lausanne). 2020 Jun 19;7:288. doi: 10.3389/fmed.2020.00288 (PMC7317305; doi:10.3389/fmed.2020.00288)
Supplement: Supplementary file 1 [file Table_1.docx]

**Suppl. Table 1**

**Univariate Cox regression analysis of factors associated**

**with secukinumab discontinuation**

| **Variable** | **Univariate analysis** | | | |
| --- | --- | --- | --- | --- |
|  | **Hazard ratio** | **95% CI** | | **p** |
|  |  | **Lower** | **Upper** |  |
| **Sex, female** | 0.68 | 0.23 | 1.96 | 0.47 |
| **Age** | 1.002 | 0.97 | 1.03 | 0.91 |
| **BMI** | 1.06 | 0.99 | 1.14 | 0.07 |
| **Smoking** | 0.87 | 0.34 | 2.20 | 0.76 |
| **Disease duration** | 1.003 | 0.95 | 1.07 | 0.72 |
| **Axial involvement** | 0.68 | 0.31 | 1.47 | 0.33 |
| **HAQ (baseline)** | 1.34 | 0.35 | 5.11 | 0.66 |
| **CRP (baseline)** | 0.98 | 0.92 | 1.05 | 0.53 |
| **DAPSA (baseline)** | 0.99 | 0.97 | 1.02 | 0.62 |
| **RDCI** | 1.10 | 0.89 | 1.36 | 0.37 |
| **Non-biologic co-administration** | 1.16 | 0.54 | 2.51 | 0.67 |
| **Glucocorticoid co-administration** | 2.91 | 1.35 | 6.28 | 0.06 |

CI: Confidence Intervals, BMI: Body Mass Index, HAQ: Health Assessment Questionnaire, CRP: C-Reactive Protein, DAPSA: Disease Activity in Psoriatic Arthritis Score, RDCI: Rheumatic Diseases Comorbidity Index
